# Differential asthma odds following respiratory infection in children from three minority populations

**DOI:** 10.1371/journal.pone.0231782

**Published:** 2020-05-05

**Authors:** Eric M. Wohlford, Luisa N. Borrell, Jennifer R. Elhawary, Brian Plotkin, Sam S. Oh, Thomas J. Nuckton, Celeste Eng, Sandra Salazar, Michael A. LeNoir, Kelley Meade, Harold J. Farber, Denise Serebrisky, Emerita Brigino-Buenaventura, William Rodriguez-Cintron, Rajesh Kumar, Shannon Thyne, Max A. Seibold, José R. Rodríguez-Santana, Esteban G. Burchard

**Affiliations:** 1 Department of Medicine, University of California, San Francisco, CA, United States of America; 2 Department of Epidemiology & Biostatistics, Graduate School of Public Health & Health Policy, City University of New York, New York, NY, United States of America; 3 Bay Area Pediatrics, Oakland, CA, United States of America; 4 Children's Hospital and Research Center Oakland, Oakland, CA, United States of America; 5 Department of Pediatrics, Section of Pulmonology, Baylor College of Medicine and Texas Children’s Hospital, Houston, TX, United States of America; 6 Pediatric Pulmonary Division, Jacobi Medical Center, Bronx, NY, United States of America; 7 Department of Allergy and Immunology, Kaiser Permanente-Vallejo Medical Center, Vallejo, CA, United States of America; 8 Veterans Caribbean Health Care System, San Juan, Puerto Rico, United States of America; 9 The Ann and Robert H. Lurie Children’s Hospital of Chicago, Chicago, IL, United States of America; 10 Department of Pediatrics, University of California, Los Angeles, Los Angeles, CA, United States of America; 11 National Jewish Health, Denver, CO, United States of America; 12 Centro de Neumologia Pediátrica, Caguas, Puerto Rico, United States of America; 13 Department of Bioengineering and Therapeutic Sciences, University of California, San Francisco, CA, United States of America; Center for Disease Control and Prevention, UNITED STATES

## Abstract

**Rationale:**

Severe early-life respiratory illnesses, particularly those caused by respiratory syncytial virus (RSV) and human rhinovirus (HRV), are strongly associated with the development of asthma in children. Puerto Rican children in particular have a strikingly high asthma burden. However, prior studies of the potential associations between early-life respiratory illnesses and asthma in Puerto Rican and other minority populations have been limited.

**Objectives:**

We sought to determine whether early-life respiratory illness was associated with asthma in Puerto Rican, Mexican American, and African American children.

**Methods:**

Using a logistic regression analysis, we examined the association between early-life respiratory illnesses (report of upper respiratory infection (URI), pneumonia, bronchitis, and bronchiolitis/RSV) within the first two years of life and physician-diagnosed asthma after the age of two in a large cohort of Puerto Rican, Mexican American, and African American children.

**Measurements and main results:**

While early-life respiratory illnesses were associated with greater asthma odds in Puerto Ricans, Mexican Americans, and African Americans, these associations were stronger among Puerto Rican children. Specifically, in Puerto Ricans, the odds was 6.15 (95% CI: 4.21–9.05) if the child reported at least one of the following respiratory illness: URI, pneumonia, bronchitis or bronchiolitis. The odds were also higher in Puerto Ricans when considering these conditions separately.

**Conclusions:**

We observed population-specific associations between early-life respiratory illnesses and asthma, which were especially significant and stronger in Puerto Ricans. Taken together with the known high burden of RSV in Puerto Rico, our results may help explain the high burden of asthma in Puerto Ricans.

## Introduction

Asthma is the most common chronic disease in children [1, 2], with genetic, environmental, and infectious risk factors. [3–5] Though the global burden of asthma is increasing, certain racial/ethnic and geographic populations are at especially high risk. Puerto Ricans are among the most severely affected populations in the world. [4] Approximately 36.5% of Puerto Ricans report they currently or previously had asthma, compared to 9.4% of African Americans, 7.6% of non-Hispanic whites, and 7.5% of Mexican Americans. [6, 7] These striking differences extend to asthma morbidity and mortality, which are 2.4- and 4-fold higher in Puerto Ricans compared to whites, respectively. [6, 7] Asthma is a complex disease and often presents as a function of genetic, environmental, and socio-cultural risk factors. [8–13] Yet asthma, and more specifically asthma disparities, are still not well understood. We propose to examine a single, but significant, facet of asthma that may explain differences in asthma prevalence across minority populations: early-life respiratory illnesses.

**Fig 1:**
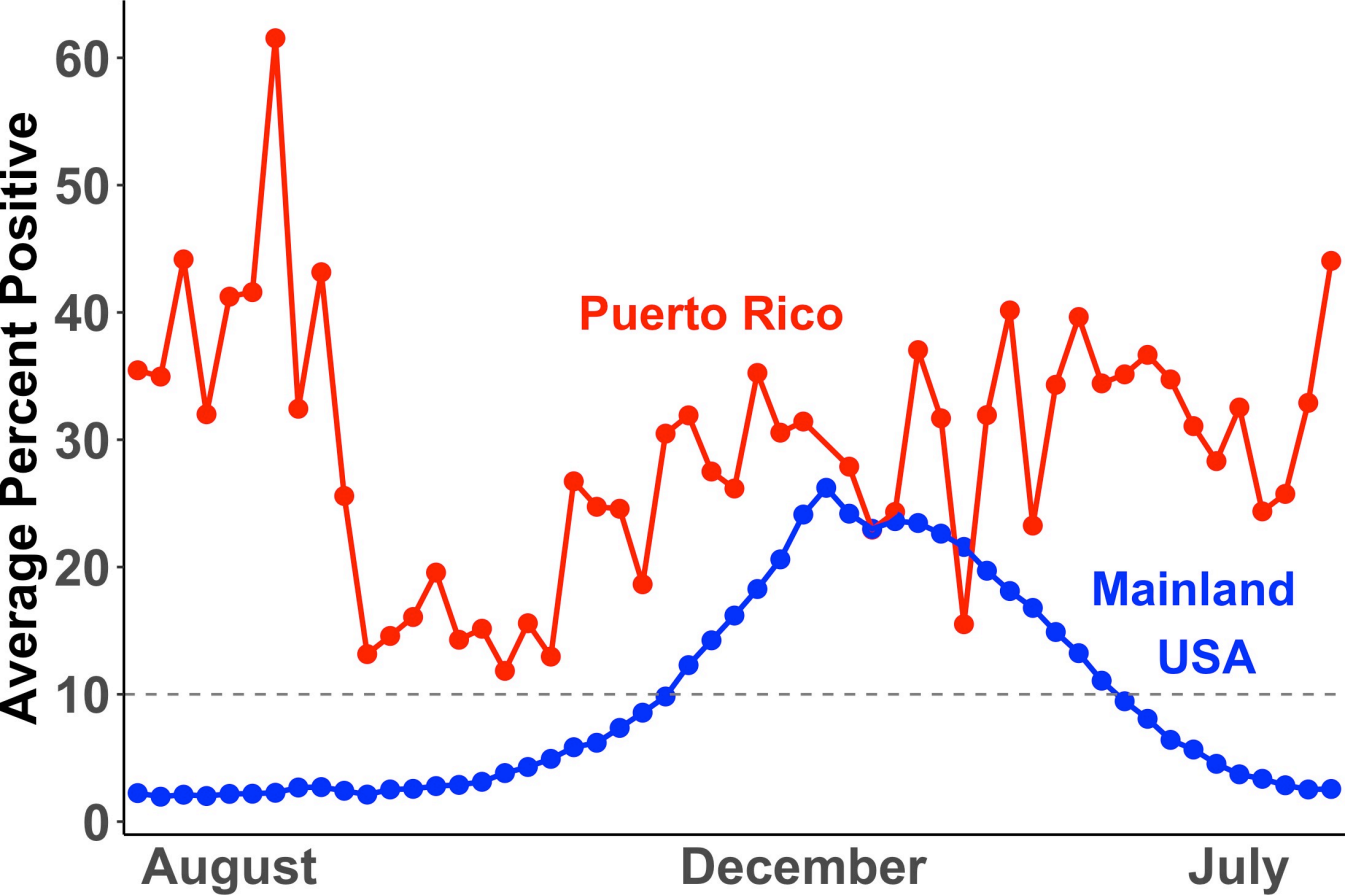
Respiratory syncytial virus (RSV) season in Puerto Rico (red) and the mainland United States (blue). RSV season begins when 10% or more of RSV tests are positive. Data shown are a simplified representation of the data obtained from McGuinness et al. Pediatr Infect Dis J. 2014. [29]

Several epidemiological studies have established a strong association between the development of childhood asthma or recurrent wheeze with exposure to severe, early-life respiratory illnesses across populations. [14–24] Associations with asthma were strongest for infections caused by respiratory syncytial virus (RSV) [14, 17, 20–24] and human rhinovirus (HRV), leading to a 2.6 and 9.8 fold probability of asthma, respectively. [18–20] Before one year of age, persistent wheezing illness is most commonly caused by RSV, which later changes to HRV in older children. [25] Both RSV and HRV are known to cause bronchiolitis, which can be a severe respiratory infection in children and is linked to later asthma development. [22, 26] These viruses have a complex interaction between genetics and environmental exposures in determining risks for asthma and related outcomes. [27, 28] Additionally, Puerto Rico has an RSV season that is year-round whereas the mainland United States only reports a 20-week season (**Fig 1**). [29, 30]

It is unclear at this time if differential responses to early-life respiratory illnesses contribute to the striking asthma disparities seen across minority populations. It is possible that genetic predisposition, environmental influences, and early-life respiratory illness work together to increase asthma susceptibility in high-risk populations. Our aim was to investigate the association of early-life respiratory illnesses with asthma susceptibility seen in our large and well-phenotyped cohort of minority children. In light of disparate disease prevalence, [6, 29, 30] we examined this association in each population (Puerto Ricans, African Americans and Mexican Americans), separately and combined. We hypothesize that differences in the prevalence of clinically diagnosed early-life respiratory infections such as upper respiratory infections (URI), pneumonia, bronchitis, and bronchiolitis across populations may be associated with asthma prevalence and further these associations may vary across these three minority population.

## Methods

### Study population

The study included participants recruited through two studies: the Genes-Environments and Admixture in Latino Americans (GALA II) study and the Study of African Americans, Asthma, Genes and Environments (SAGE), described in detail elsewhere. [10, 13, 31] Briefly, GALA II and SAGE are parallel case-control studies of asthma conducted between 2006 to 2014 in Latino (Mexican American and Puerto Rican) and African American children aged 8–21, respectively. SAGE participants were recruited from the San Francisco Bay Area and GALA II participants were recruited from across the continental United States (Chicago, Houston, New York City, and the San Francisco Bay Area) and Puerto Rico. Questionnaires were administered to adult participants and parents of minors. All participants provided written consent to being in the study. Consent was obtained from all adult participants and parent/legal guardians of minor participants. The study protocols for both GALA II and SAGE were approved by the UCSF Human Research Protection Program Institutional Review Board (IRB) and all institutions participating in recruitment obtained the appropriate approvals from their IRBs for recruitment related activity.

Out of a total of 6,023, participants with asthma onset prior to the age of two (n = 1,450) and those individuals without complete illness and covariate data (n = 2,301) were excluded from the study. Those diagnosed with asthma before age two were initially excluded from analysis to better delineate any causal association between early-life respiratory illness before age two and subsequent diagnosis of asthma. The exclusion criteria yielded an analytical sample of 2,824 subjects, including both cases (n = 1,091) and controls (n = 1,733).

### Outcomes

The outcome of our study was physician diagnosed asthma diagnosed after the age of two reported by the parent. [31] Eligible control subjects had no reported history of asthma, lung disease, or chronic illness, and no reported symptoms of coughing, wheezing, or shortness of breath in the two years before enrollment.

### Exposure

The exposure was parental report of physician diagnosed early-life respiratory illnesses within the first two years of life. Early-life respiratory illnesses included URI, pneumonia, bronchitis, and bronchiolitis/RSV, which were analyzed separately. In addition, the report of at least one of the four previous illnesses was used to categorize children with “any respiratory illness” and none otherwise. As this was a retrospective study, we could not rely on pathogen identification as a means to confirm the viral species present during the illness.

### Covariates

Consistent with previous studies, [32, 33] we considered factors known to be associated with early-life respiratory illnesses and with asthma. These included sex, underweight at birth (yes/no), maternal smoking during pregnancy (yes/no), whether the subject was breastfed (yes/no), number of older siblings (none, one, two or more), socioeconomic status (SES; low, medium, high; described further in **S1 Text**), and recruitment site. However, recruitment site was not included in the African American models because all the participants were recruited from one site only.

### Statistical analysis

Descriptive statistics were calculated for the overall study population and for each minority population separately. We used logistic regression to quantify the association between physician-diagnosed asthma and self-reported respiratory illnesses in the first two years of life in all three populations (Puerto Ricans, Mexican Americans, and African Americans) combined. In addition to sex, underweight at birth, maternal smoking during pregnancy, breastfeeding, number of older siblings, SES and recruitment site, we adjusted for the underlying substructure of populations in the dataset using global African and European ancestry estimates (described further in **S2 Text**) as a proxy. Because race/ethnicity may act as an effect modifier due to the large differences in asthma prevalence, [6, 7] we estimated this association stratified by race/ethnicity. Significance for main effects was determined at *p* < 0.05. The statistical programming language R version 3.5.1 was used to perform all analyses.

### Data availability

Biological, environmental and phenotypic data analyzed in the current study are available in the dbGAP repository (study accession numbers: SAGE phs00921.v1.p1, GALA II phs001180.v1.p1). Psychosocial data analyzed in the current study (experience of discrimination and socioeconomic status) are not publicly available due to the sensitive nature of the data and privacy concerns for study participants. Psychosocial data is currently stored in the UCSF Box repository and is available upon reasonable request at asthma.collaboratory@ucsf.edu.

## Results

**Fig 2:**
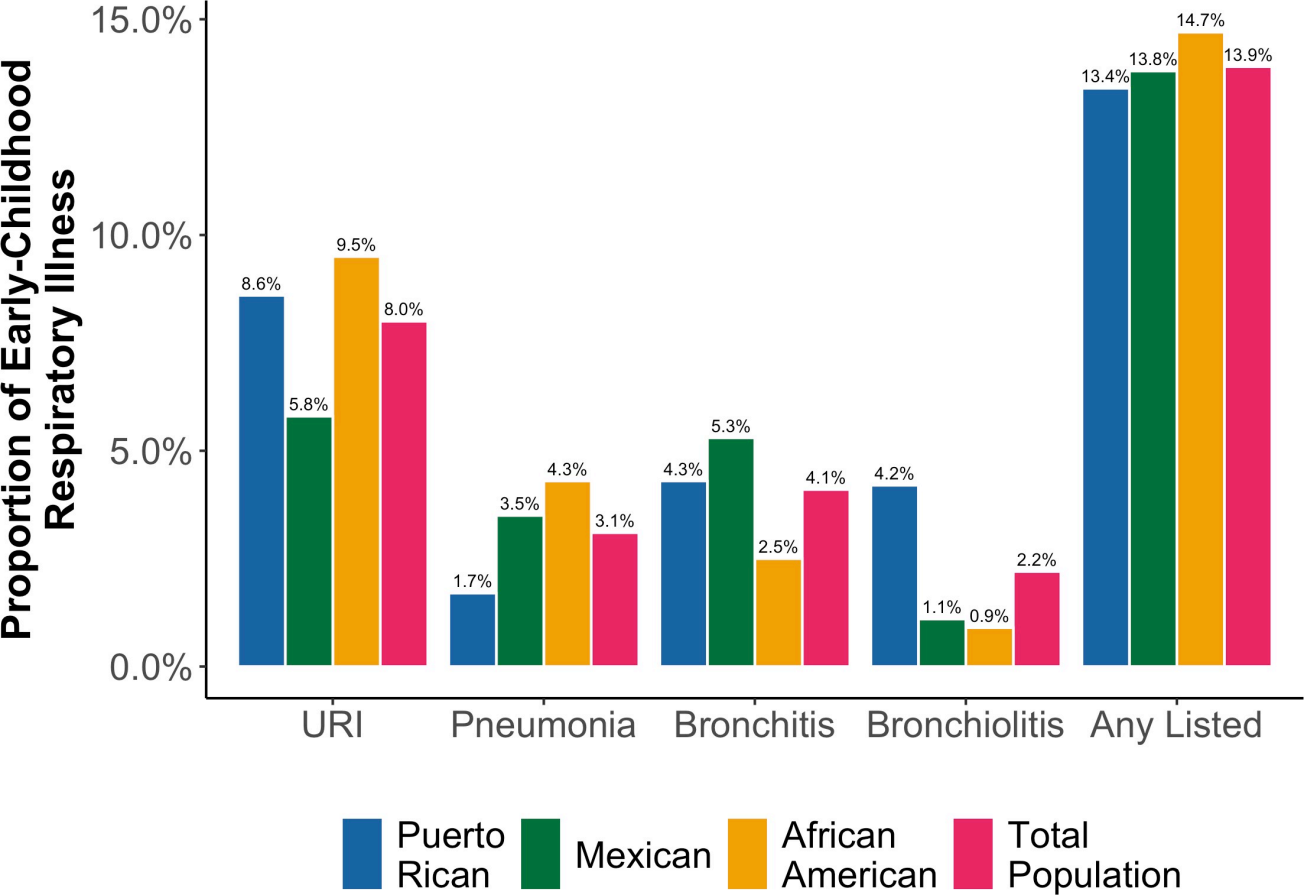
Population-specific proportions of selected respiratory illnesses during the first 2 years of life for our study population.

Descriptive characteristics of our final study population are presented in **Table 1**. When compared with children without asthma, those with asthma were more likely to be male, to have a mother who smoked during pregnancy, and have lower SES. These distributions were observed in our sample regardless of race/ethnicity with a few exceptions. For instance, while Puerto Rican and Mexican American children without asthma were more likely to be breastfed than their counterparts with asthma, the opposite was true for African Americans. Additionally, we found that Puerto Ricans were 4- to 9-fold more likely to report an RSV infection or bronchiolitis in the first two years of life than Mexican Americans and African Americans, respectively (**Fig 2**).

**Table 1:** Descriptive statistics for selected characteristics for GALA II and SAGE participants: 2006–2014.

	Puerto Rican				Mexican American				African American				Total Population			
	*Case*		*Control*		*Case*		*Control*		*Case*		*Control*		*Case*		*Control*	
	*N*	*%*	*N*	*%*	*N*	*%*	*N*	*%*	*N*	*%*	*N*	*%*	*N*	*%*	*N*	*%*
Number of subjects	284	100	783	100	375	100	504	100	432	100	446	100	1091	100	1733	100
Males	150	52.8	361	46.1	212	56.5	210	41.7	218	50.5	187	41.9	580	53.2	758	43.7
Underweight at birth	32	11.3	61	7.8	24	6.4	35	6.9	36	8.3	45	10.1	92	8.4	141	8.1
In-utero smoke exposure	14	4.9	40	5.1	14	3.7	9	1.8	78	18.1	54	12.1	106	9.7	103	5.9
Breastfed	141	49.6	432	55.2	285	76	401	79.6	262	60.6	244	54.7	688	63.1	1077	62.1
Number of older siblings																
*0*	74	26.1	200	25.5	132	35.2	195	38.7	247	57.2	208	46.6	453	41.5	603	34.8
*1*	92	32.4	274	35	138	36.8	176	34.9	101	23.4	132	29.6	331	30.3	582	33.6
*2 or more*	118	41.5	309	39.5	105	28	133	26.4	84	19.4	106	23.8	307	28.1	548	31.6
Socioeconomic status*																
*High*	89	31.3	271	34.6	114	30.4	116	23	163	37.7	160	35.9	366	33.5	547	31.6
*Medium*	51	18	136	17.4	79	21.1	84	16.7	113	26.2	137	30.7	243	22.3	357	20.6
*Low*	144	50.7	376	48	182	48.5	304	60.3	156	36.1	149	33.4	482	44.2	829	47.8
Recruitment site																
*Chicago*	18	6.3	23	2.9	137	36.5	180	35.7	-	-	-	-	155	14.2	203	11.7
*Houston*	1	0.4	-	-	96	25.6	91	18.1	-	-	-	-	97	8.9	91	5.3
*New York*	27	9.5	34	4.3	20	5.3	64	12.7	-	-	-	-	47	4.3	98	5.7
*San Francisco Bay Area*	1	0.4	1	0.1	122	32.5	169	33.5	432	100	446	100	555	50.9	616	35.5
*Puerto Rico*	237	83.5	725	92.6	-	-	-	-	-	-	-	-	237	21.7	725	41.8
URI	57	20.1	35	4.5	24	6.4	27	5.4	67	15.5	16	3.6	148	13.6	78	4.5
Pneumonia	13	4.6	5	0.6	20	5.3	11	2.2	24	5.6	14	3.1	57	5.2	30	1.7
Bronchitis	36	12.7	10	1.3	37	9.9	10	2	16	3.7	6	1.3	89	8.2	26	1.5
Bronchiolitis/RSV	31	10.9	14	1.8	6	1.6	4	0.8	6	1.4	2	0.4	43	3.9	20	1.2
Any Listed	89	31.3	54	6.9	75	20	46	9.1	98	22.7	31	7	262	24	131	7.6

*Socioeconomic status was derived from a combination of mother’s education level, health insurance status, and household income weighted by region, see **S1 Text** for more information.

**Fig 3:**
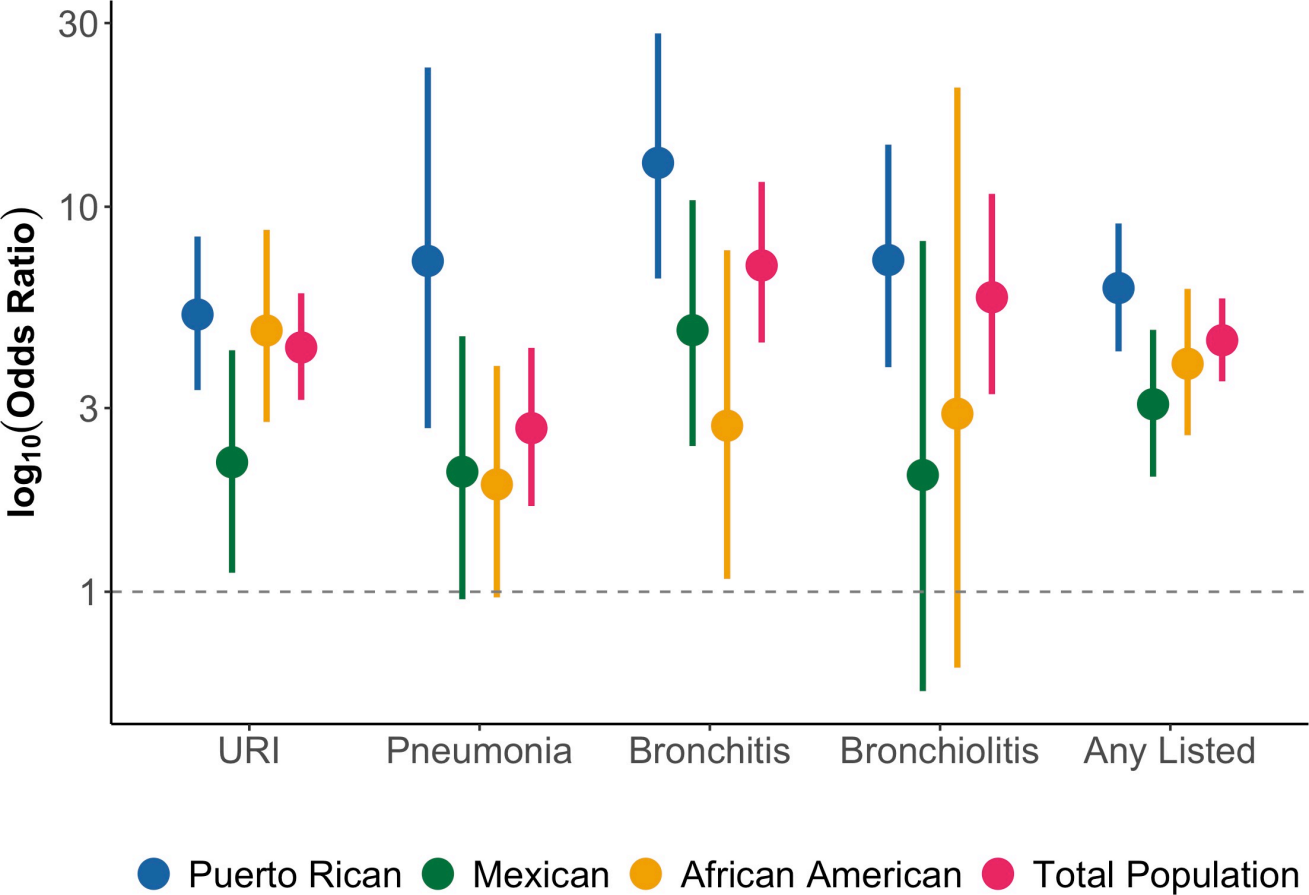
Odds ratios plotted on a log scale of the association between early-life respiratory illnesses before the age of two and asthma diagnosis after the age of two according to race/ethnicity and in the total population.

In our total population, we observed that the odds of asthma was 4.31 (95% CI: 3.15–5.96) for URI, 2.66 (95% CI: 1.67–4.30) for pneumonia, 7.04 (95% CI: 4.44–11.60) for bronchitis, 5.82 (95% CI: 3.26–10.80) for bronchiolitis/RSV, and 4.50 (95% CI: 3.52–5.78) if the participant reported having at least one early-life respiratory illness (**Table 2 and Fig 3**). In the population-specific stratified analyses, we also observed that the odds of asthma in Puerto Ricans was 5.25 (95% CI: 3.34–8.37) for URI, 7.23 (95% CI: 2.66–23.0) for pneumonia, 13.0 (95% CI: 6.51–28.20) for bronchitis, 7.27 (95% CI: 3.83–14.50) for bronchiolitis/RSV, and 6.15 (95% CI: 4.21–9.05) if the participant reported having at least one early-life respiratory illness (**Table 2 and Fig 3**). Similarly, and with few exceptions, we observed significant associations between early-life respiratory illness and asthma in Mexican American and in African American children. However, these associations were smaller in magnitude than the ones observed among Puerto Ricans. Specifically, the odds of asthma in Mexican Americans and African Americans, respectively, were 2.17 (95% CI: 1.12–4.24) and 4.77 (95% CI: 2.76–8.71) for URI, 2.05 (95% CI: 0.96–4.61) and 1.90 (95% CI: 0.97–3.86) for pneumonia, 4.78 (95% CI: 2.39–10.40) and 2.70 (95% CI: 1.08–7.71) for bronchitis, 2.01 (95% CI: 0.55–8.15) and 2.90 (95% CI: 0.64–20.40) for bronchiolitis/RSV, and 3.07 (95% CI: 1.99–4.79) and 3.91 (95% CI: 2.55–6.12) if the participant reported having at least one early-life respiratory illness (**Table 2 and Fig 3**).

**Table 2:** Adjusted* odds ratios and confidence intervals for the association analysis between respiratory illnesses in the first two years of life and physician-diagnosed asthma after the age of two according to race/ethnicity and in the total population in GALA II and SAGE: 2006–2014.

	Puerto Rican (n = 1,067)	Mexican American (n = 879)	African American (n = 878)	Total Population (n = 2,824)
URI	5.25 (3.34–8.37)	2.17 (1.12–4.24)	4.77 (2.76–8.71)	4.31 (3.15–5.96)
Pneumonia	7.23 (2.66–23.0)	2.05 (0.96–4.61)	1.90 (0.97–3.86)	2.66 (1.67–4.30)
Bronchitis	13.0 (6.51–28.20)	4.78 (2.39–10.40)	2.70 (1.08–7.71)	7.04 (4.44–11.60)
Bronchiolitis/RSV	7.27 (3.83–14.50)	2.01 (0.55–8.15)	2.90 (0.64–20.40)	5.82 (3.26–10.80)
Any Listed	6.15 (4.21–9.05)	3.07 (1.99–4.79)	3.91 (2.55–6.12)	4.50 (3.52–5.78)

*Adjusted for sex, underweight at birth, maternal smoking during pregnancy, breastfeeding, number of older siblings, SES, recruitment site and global ancestry. Recruitment site was not included in the African American models as they were recruited from one site only.

To test statistically significant differences in odds ratios between racial/ethnic groups, we compared the beta coefficients using t-tests. [34] We found that there were significant differences (*p* < 0.05) between Puerto Ricans and Mexican Americans for URI and Any Listed, and between Puerto Ricans and African Americans for bronchitis and pneumonia (**S1 Table**).

In a separate analysis, we examined these associations including those individuals diagnosed with asthma before the age of two (**S2 Table**). Our results showed that all early-life respiratory illnesses were significantly associated (*p* < 0.05) with asthma diagnosis in our population and regardless of race/ethnicity, with the exception of RSV in Mexican Americans (**S3 Table**). It is worth noting that we observed significantly higher odds of asthma in Puerto Ricans than in Mexican Americans for every respiratory infection examined (**S4 Table**).

We additionally compared the odds of asthma after an early-life respiratory illness in Puerto Ricans living in Puerto Rico (Islanders) with those living in the mainland United States (Mainlanders); descriptive characteristics for these populations can be found in **S5 Table**. We found high asthma odds following early-life respiratory illness in Islanders relative to Mainlanders (**S6 Table**) though due to the small number of Mainlanders we were underpowered to detect statistically significant differences between these groups.

## Discussion

Overall, we found that early-life respiratory illnesses such as URI, pneumonia, bronchitis, and bronchiolitis/RSV are significantly associated with asthma diagnosis after the age of two regardless of race/ethnicity. However, Puerto Ricans had the strongest associations.

The association between early-life respiratory illnesses and asthma has been well documented. [14–24] Yet, little is known about racial/ethnic differences observed for the associations between early-life respiratory illnesses and the development of asthma later on in childhood.

Previous research on the association between early-life respiratory infection and the development of asthma has yielded conflicting results. Having a wheezing illness due to RSV or HRV infection in early life has previously shown to be associated with a 2.6- and 9.8-fold increase, respectively, in asthma odds by age six in a mostly white population. [18] Additionally, a recent study found that the severity of the RSV infection was strongly associated with childhood wheezing at age five in a mostly white population. [17] However, the vast majority of children infected by respiratory viruses like RSV do not go on to develop respiratory illnesses like recurrent wheezing and asthma. [33] Currently, it is unclear why only a minority of children develop asthma after exposure to an early-life respiratory illness. One plausible explanation is that these respiratory illnesses alter the airway in early life, which leads to asthma later on in childhood. In fact, previous studies have shown that in adults with asthma, the airway epithelium is altered in a heterogeneous manner. [35] Another possibility is that children who are already genetically or environmentally prone to asthma present with early-life respiratory illness as an early manifestation of asthma. Indeed, previous studies have shown asymptomatic carriage of RSV and rhinovirus in children, [36–38] suggesting a spectrum of disease which may be affected by underlying asthma predisposition. [39, 40] Our findings show greater asthma odds associated with early-life respiratory illnesses in all populations studied, but that the odds was stronger in the Puerto Rican population. These findings are consistent with previous studies [40–42] showing that early-life respiratory illnesses are significantly associated with the development of asthma and asthma-related outcomes while additionally adding that these associations may vary by region and/or population.

Most children who develop respiratory viral infections in early life experience minor illness, but some develop much more severe illnesses that involve lower respiratory symptoms like wheezing. [15, 16, 36] These more severe wheezing illnesses at an early stage in life are associated with a high risk for recurrent wheezing and asthma later in childhood, [18, 19] particularly if these wheezing illnesses were caused by RSV or HRV. [14, 17, 21–24] Puerto Rican children are especially at risk due to the year-round seasonality of RSV infections. [29, 30] In fact, 1,406 cases of bronchiolitis/RSV were reported in children with a mean age of seven months from six Puerto Rican hospitals over a period of nine months. [39] Our findings showed that not only is the prevalence of bronchiolitis/RSV infection highest in Puerto Rican children in our study population, but that the odds for asthma among children who have experienced a bronchiolitis/RSV infection in early life was the highest for Puerto Rican Islanders. These observations suggest that the burden of childhood asthma after bronchiolitis/RSV is particularly high Puerto Rican children. While it is known that early-life respiratory infections are associated with asthma, our findings show racial/ethnic disparities for the odds of asthma post-infection in our population, with Puerto Rican children carrying the greater burden of asthma.

In our study of children diagnosed with asthma after two years of age, some confidence intervals were too wide to make definitive conclusions regarding the odds ratios for asthma after infection in different racial/ethnic groups. However, when we included those children diagnosed with asthma before two years of age, we were able to better delineate the association between different respiratory infections and asthma development across racial/ethnic groups. Interestingly Puerto Ricans and Mexican Americans are both considered Hispanic/Latino for pulmonary function testing and other health outcomes. However, these populations had widely divergent prevalence of asthma after early-life respiratory infections. Puerto Ricans consistently had higher odds of asthma after all respiratory infections studied. Whether the observed association between early-life respiratory infection and greater asthma odds is causal of the alarmingly high prevalence of asthma in Puerto Ricans requires further study.

While we are not able to draw definitive conclusions about the greater odds of asthma after respiratory infection in Puerto Rican Islanders versus Mainlanders due to the limited number of mainland US Puerto Rican children studied, we find it interesting that Mainlander Puerto Ricans tended to have a lower odds of asthma after respiratory infections relative to Islanders. This may suggest an environmental effect associated with living in Puerto Rico that affects the prevalence of asthma. Respiratory infections likely represent one of several factors leading to increased asthma in Islander Puerto Ricans relative to Mainlanders, which may include other environmental and socio-cultural factors. Further studies examining this associations in Puerto Ricans living in the island and in the mainland US can help clarify this observation.

Our study may have been limited because the majority (71%) of Puerto Ricans in the GALA study were diagnosed with asthma before the age of two. In contrast, only 35% of Mexican Americans, and 50% of African Americans were diagnosed with asthma before two years old. Our secondary analysis including children diagnosed with asthma before two years of age addressed these differences. Additionally, our exposure measurements were determined retrospectively, which may introduce biases in the data. Specifically, recall bias may affect the accuracy of answers to questions about events in the distant past. However, if bias were to occur, this bias will be non-differential and affect all racial/ethnic groups studied, biasing the results towards the null. This study was underpowered to detect any significant associations of early-life respiratory illnesses on asthma in Puerto Rican Mainlanders versus Puerto Rican Islanders. Thus, we were not able to determine whether the year-round RSV prevalence in Puerto Rico influenced the association between respiratory illnesses and asthma. However, our study suggests that there are clear population-specific differences in asthma susceptibility across a variety of respiratory illnesses using a large cohort of minority children from three distinct racial/ethnic populations. Moreover, our study also benefits from the wide range of clinical, social, and genetic data available on these children.

## Conclusion

Early-life respiratory illnesses such as RSV, which is highly prevalent in Puerto Ricans, have previously been associated with asthma. Our findings indicate that early-life respiratory infections are particularly associated with the later development of asthma in our population regardless of race/ethnicity, with Puerto Rican children having the stronger associations.

## Supporting information

S1 Text Derivation of socioeconomic status (SES). (DOCX)

S2 Text Derivation of global ancestry estimates. (DOCX)

S1 Table Pairwise comparison of odds ratios for asthma after two years of age following early-life respiratory infection by racial/ethnic group. *Definition of Abbreviations*: URI = Upper Respiratory Infection.
(DOCX)

S2 Table Descriptive statistics for selected characteristics of the study population including those diagnosed with asthma before age two (N = 3,722). (DOCX)

S3 Table Odds ratios and confidence intervals from the population-specific and combined association analysis between respiratory illnesses in the first two years of life and physician-diagnosed asthma according to race/ethnicity and in the total population in GALA II and SAGE: 2006–2014. *Definition of Abbreviations*: URI = Upper Respiratory Infection.
(DOCX)

S4 Table Pairwise comparison of odds ratios for asthma following early-life respiratory infection by racial/ethnic group including those diagnosed with asthma before age two. (DOCX)

S5 Table Descriptive characteristics of Puerto Rican Islanders and Mainlanders. (DOCX)

S6 Table Odds ratios and confidence intervals from the geography-specific analysis between respiratory illnesses in the first two years of life and physician-diagnosed asthma after the age of two in the two Puerto Rican populations: Islanders and Mainlanders. *Definition of Abbreviations*: URI = Upper Respiratory Infection.
(DOCX)

## Abbreviations

RSV: respiratory syncytial virus
HRV: human rhinovirus
GALA II: Genes-Environments and Admixture in Latino Americans
SAGE: The Study of African Americans, Asthma, Genes and Environments
URI: upper respiratory infection
SES: socioeconomic status

## References

[1] Asthma. World Health Organization (WHO). Available from: http://www.who.int/respiratory/asthma/en/

[2] Bloom B, Cohen RA, Freeman G. Summary health statistics for U.S. children: National Health Interview Survey, 2009. Vital Health Stat 10. 2010;(247):1–82. Epub 2011/05/14. . 21563639

[3] Aligne CA, Auinger P, Byrd RS, Weitzman M. Risk factors for pediatric asthma. Contributions of poverty, race, and urban residence. Am J Respir Crit Care Med. 2000;162(3 Pt 1):873–7. Epub 2000/09/16. 10.1164/ajrccm.162.3.9908085 . 10988098

[4] Oh SS, White MJ, Gignoux CR, Burchard EG. Making Precision Medicine Socially Precise. Take a Deep Breath. Am J Respir Crit Care Med. 2016;193(4):348–50. 10.1164/rccm.201510-2045ED 26871667 PMC4803087

[5] Subbarao P, Mandhane PJ, Sears MR. Asthma: epidemiology, etiology and risk factors. CMAJ. 2009;181(9):E181–90. Epub 2009/09/16. 10.1503/cmaj.080612 19752106 PMC2764772

[6] Barr RG, Aviles-Santa L, Davis SM, Aldrich TK, Gonzalez F, 2nd, Henderson AG, et al Pulmonary Disease and Age at Immigration among Hispanics. Results from the Hispanic Community Health Study/Study of Latinos. Am J Respir Crit Care Med. 2016;193(4):386–95. Epub 2015/10/10. 10.1164/rccm.201506-1211OC 26451874 PMC4803083

[7] Akinbami LJ. Asthma Prevalence, Health Care Use and Mortality: United States, 2003–05: Center for Disease Control and Prevention: National Center for Health Statistics; 2010. Available from: https://www.cdc.gov/nchs/data/hestat/asthma03-05/asthma03-05.htm

[8] Neophytou AM, Oh SS, Hu D, Huntsman S, Eng C, Rodríguez-Santana JR, et al In utero tobacco smoke exposure, DNA methylation, and asthma in Latino children. Environ Epidemiol. 2019;3(3):e048 Epub 2019/06/19. 10.1097/EE9.0000000000000048 31342008 PMC6571182

[9] Neophytou AM, White MJ, Oh SS, Thakur N, Galanter JM, Nishimura KK, et al Air Pollution and Lung Function in Minority Youth with Asthma in the GALA II (Genes-Environments and Admixture in Latino Americans) and SAGE II (Study of African Americans, Asthma, Genes, and Environments) Studies. Am J Respir Crit Care Med. 2016;193(11):1271–80. Epub 2016/01/07. 10.1164/rccm.201508-1706OC 26734713 PMC4910900

[10] Oh SS, Tcheurekdjian H, Roth LA, Nguyen EA, Sen S, Galanter JM, et al Effect of secondhand smoke on asthma control among black and Latino children. J Allergy Clin Immunol. 2012;129(6):1478–83 e7. Epub 2012/05/04. 10.1016/j.jaci.2012.03.017 22552109 PMC3367092

[11] Pino-Yanes M, Thakur N, Gignoux CR, Galanter JM, Roth LA, Eng C, et al Genetic ancestry influences asthma susceptibility and lung function among Latinos. J Allergy Clin Immunol. 2015;135(1):228–35. 10.1016/j.jaci.2014.07.053 25301036 PMC4289103

[12] Thakur N, Barcelo NE, Borrell LN, Singh S, Eng C, Davis A, et al Perceived Discrimination Associated With Asthma and Related Outcomes in Minority Youth: The GALA II and SAGE II Studies. Chest. 2017;151(4):804–12. Epub 2016/12/06. 10.1016/j.chest.2016.11.027 27916618 PMC5472516

[13] Thakur N, Oh SS, Nguyen EA, Martin M, Roth LA, Galanter J, et al Socioeconomic status and childhood asthma in urban minority youths. The GALA II and SAGE II studies. Am J Respir Crit Care Med. 2013;188(10):1202–9. 10.1164/rccm.201306-1016OC 24050698 PMC3863734

[14] Bacharier LB, Cohen R, Schweiger T, Yin-Declue H, Christie C, Zheng J, et al Determinants of asthma after severe respiratory syncytial virus bronchiolitis. J Allergy Clin Immunol. 2012;130(1):91–100 e3. Epub 2012/03/27. 10.1016/j.jaci.2012.02.010 22444510 PMC3612548

[15] Castro-Rodriguez JA, Forno E, Rodriguez-Martinez CE, Celedon JC. Risk and Protective Factors for Childhood Asthma: What Is the Evidence? J Allergy Clin Immunol Pract. 2016;4(6):1111–22. Epub 2016/06/12. 10.1016/j.jaip.2016.05.003 27286779 PMC5107168

[16] Castro-Rodriguez JA, Holberg CJ, Wright AL, Martinez FD. A clinical index to define risk of asthma in young children with recurrent wheezing. Am J Respir Crit Care Med. 2000;162(4 Pt 1):1403–6. Epub 2000/10/13. 10.1164/ajrccm.162.4.9912111 . 11029352

[17] Escobar GJ, Masaquel AS, Li SX, Walsh EM, Kipnis P. Persistent recurring wheezing in the fifth year of life after laboratory-confirmed, medically attended respiratory syncytial virus infection in infancy. BMC Pediatr. 2013;13:97 Epub 2013/06/21. 10.1186/1471-2431-13-97 23782528 PMC3703269

[18] Jackson DJ, Gangnon RE, Evans MD, Roberg KA, Anderson EL, Pappas TE, et al Wheezing rhinovirus illnesses in early life predict asthma development in high-risk children. Am J Respir Crit Care Med. 2008;178(7):667–72. Epub 2008/06/21. 10.1164/rccm.200802-309OC 18565953 PMC2556448

[19] Kusel MM, de Klerk NH, Kebadze T, Vohma V, Holt PG, Johnston SL, et al Early-life respiratory viral infections, atopic sensitization, and risk of subsequent development of persistent asthma. J Allergy Clin Immunol. 2007;119(5):1105–10. Epub 2007/03/14. 10.1016/j.jaci.2006.12.669 . 17353039 PMC7125611

[20] Lemanske RF Jr., Jackson DJ, Gangnon RE, Evans MD, Li Z, Shult PA, et al Rhinovirus illnesses during infancy predict subsequent childhood wheezing. J Allergy Clin Immunol. 2005;116(3):571–7. Epub 2005/09/15. 10.1016/j.jaci.2005.06.024 . 16159626

[21] Sigurs N. Epidemiologic and clinical evidence of a respiratory syncytial virus-reactive airway disease link. Am J Respir Crit Care Med. 2001;163(3 Pt 2):S2–6. Epub 2001/03/20. 10.1164/ajrccm.163.supplement_1.2011109 . 11254543

[22] Sigurs N, Bjarnason R, Sigurbergsson F, Kjellman B. Respiratory syncytial virus bronchiolitis in infancy is an important risk factor for asthma and allergy at age 7. Am J Respir Crit Care Med. 2000;161(5):1501–7. Epub 2000/05/12. 10.1164/ajrccm.161.5.9906076 . 10806145

[23] Sigurs N, Bjarnason R, Sigurbergsson F, Kjellman B, Bjorksten B. Asthma and immunoglobulin E antibodies after respiratory syncytial virus bronchiolitis: a prospective cohort study with matched controls. Pediatrics. 1995;95(4):500–5. Epub 1995/04/01. . 7700748

[24] Sigurs N, Gustafsson PM, Bjarnason R, Lundberg F, Schmidt S, Sigurbergsson F, et al Severe respiratory syncytial virus bronchiolitis in infancy and asthma and allergy at age 13. Am J Respir Crit Care Med. 2005;171(2):137–41. Epub 2004/11/02. 10.1164/rccm.200406-730OC . 15516534

[25] Jartti T, Gern JE. Rhinovirus-associated wheeze during infancy and asthma development. Curr Respir Med Rev. 2011;7(3):160–6. Epub 2011/06/01. 10.2174/157339811795589423 23066381 PMC3469323

[26] Jartti T, Korppi M. Rhinovirus-induced bronchiolitis and asthma development. Pediatr Allergy Immunol. 2011;22(4):350–5. Epub 2011/05/04. 10.1111/j.1399-3038.2011.01170.x . 21535176

[27] Caliskan M, Bochkov YA, Kreiner-Moller E, Bonnelykke K, Stein MM, Du G, et al Rhinovirus wheezing illness and genetic risk of childhood-onset asthma. N Engl J Med. 2013;368(15):1398–407. Epub 2013/03/29. 10.1056/NEJMoa1211592 23534543 PMC3755952

[28] Voraphani N, Stern DA, Wright AL, Guerra S, Morgan WJ, Martinez FD. Risk of current asthma among adult smokers with respiratory syncytial virus illnesses in early life. Am J Respir Crit Care Med. 2014;190(4):392–8. Epub 2014/06/14. 10.1164/rccm.201311-2095OC 24927374 PMC4214125

[29] McGuiness CB, Boron ML, Saunders B, Edelman L, Kumar VR, Rabon-Stith KM. Respiratory syncytial virus surveillance in the United States, 2007–2012: results from a national surveillance system. Pediatr Infect Dis J. 2014;33(6):589–94. Epub 2014/01/22. 10.1097/INF.0000000000000257 24445835 PMC4025589

[30] Molinari Such M, Garcia I, Garcia L, Puig G, Pedraza L, Marin J, et al Respiratory syncytial virus-related bronchiolitis in Puerto Rico. P R Health Sci J. 2005;24(2):137–40. Epub 2005/08/25. . 16116931

[31] Nishimura KK, Galanter JM, Roth LA, Oh SS, Thakur N, Nguyen EA, et al Early-life air pollution and asthma risk in minority children. The GALA II and SAGE II studies. Am J Respir Crit Care Med. 2013;188(3):309–18. Epub 2013/06/12. 10.1164/rccm.201302-0264OC 23750510 PMC3778732

[32] Gillman MW, Barker D, Bier D, Cagampang F, Challis J, Fall C, et al Meeting report on the 3rd International Congress on Developmental Origins of Health and Disease (DOHaD). Pediatr Res. 2007;61(5 Pt 1):625–9. Epub 2007/04/07. 10.1203/pdr.0b013e3180459fcd . 17413866

[33] Pullan CR, Hey EN. Wheezing, asthma, and pulmonary dysfunction 10 years after infection with respiratory syncytial virus in infancy. Br Med J (Clin Res Ed). 1982;284(6330):1665–9. Epub 1982/06/05. 10.1136/bmj.284.6330.1665 6805648 PMC1498624

[34] Genome Toolbox. Test for a Difference in Two Odds Ratios 2014. Available from: http://genometoolbox.blogspot.com/2014/06/test-for-difference-in-two-odds-ratios.html

[35] Bai J, Smock SL, Jackson GR Jr., MacIsaac KD, Huang Y, Mankus C, et al Phenotypic responses of differentiated asthmatic human airway epithelial cultures to rhinovirus. PLoS One. 2015;10(2):e0118286 Epub 2015/02/24. 10.1371/journal.pone.0118286 25706956 PMC4338293

[36] Global Initiative for Asthma. Global Strategy for Asthma Management and Prevention 2017. Available from: www.ginasthma.org

[37] Jansen RR, Wieringa J, Koekkoek SM, Visser CE, Pajkrt D, Molenkamp R, et al Frequent detection of respiratory viruses without symptoms: toward defining clinically relevant cutoff values. J Clin Microbiol. 2011;49(7):2631–6. Epub 2011/05/06. 10.1128/JCM.02094-10 21543571 PMC3147826

[38] Munywoki PK, Koech DC, Agoti CN, Bett A, Cane PA, Medley GF, et al Frequent Asymptomatic Respiratory Syncytial Virus Infections During an Epidemic in a Rural Kenyan Household Cohort. J Infect Dis. 2015;212(11):1711–8. Epub 2015/05/06. 10.1093/infdis/jiv263 25941331 PMC4633757

[39] Matias I, Garcia I, Garcia-Fragoso L, Puig G, Pedraza L, Rodriguez L, et al Trends of Respiratory Syncytial Virus Infections in Children under 2 Years of Age in Puerto Rico. P R Health Sci J. 2015;34(2):98–101. Epub 2015/06/11. . 26061061

[40] Wennergren G, Kristjansson S. Relationship between respiratory syncytial virus bronchiolitis and future obstructive airway diseases. Eur Respir J. 2001;18(6):1044–58. Epub 2002/02/07. 10.1183/09031936.01.00254101 . 11829086

[41] Burchard EG, Ziv E, Coyle N, Gomez SL, Tang H, Karter AJ, et al The importance of race and ethnic background in biomedical research and clinical practice. N Engl J Med. 2003;348(12):1170–5. 10.1056/NEJMsb025007 . 12646676

[42] Szentpetery SE, Forno E, Canino G, Celedon JC. Asthma in Puerto Ricans: Lessons from a high-risk population. J Allergy Clin Immunol. 2016;138(6):1556–8. Epub 2016/10/19. 10.1016/j.jaci.2016.08.047 27751794 PMC5189666

