# Supplementary material for: Differential asthma odds following respiratory infection in children from three minority populations
Source: PLoS One. 2020 May 5;15(5):e0231782. doi: 10.1371/journal.pone.0231782 (PMC7199930; doi:10.1371/journal.pone.0231782)
Supplement: S1 Text — (DOCX) [file pone.0231782.s001.docx]

**Derivation of Socioeconomic status (SES)**
SES was derived from a combination of mother's education level, insurance status, and household income weighted by region of recruitment. Each component of the SES was scored on a three-point scale with 1 being lowest and 3 being the highest. Mother's education was broken down by: less than high school (1), high school diploma or equivalent (2), and greater than high school (3). Insurance status was broken down by: no insurance (1), insurance through the government (2), and insurance through self, a family member, or an employer (3). Household income was split into tertiles with recruitment region acting as a weight for income level. The three components were then averaged and partitioned into a three level categorical variable (high, medium, low) using tertile cutoff points.

**Supplemental References**

S1. Alexander DH, Lange K. Enhancements to the ADMIXTURE algorithm for individual ancestry estimation. BMC Bioinformatics. 2011;12:246.
