# Supplementary material for: Differential asthma odds following respiratory infection in children from three minority populations
Source: PLoS One. 2020 May 5;15(5):e0231782. doi: 10.1371/journal.pone.0231782 (PMC7199930; doi:10.1371/journal.pone.0231782)
Supplement: S2 Text — (DOCX) [file pone.0231782.s002.docx]

**Derivation of Global ancestry estimates (SES)**
Global estimates of the proportion of African, European, and Native American ancestry were inferred for each individual using ADMIXTURE.[S1] For Latino populations, we used a three-population model including ancestral genotypes from Europeans (CEU from HapMap), West Africans (YRI from HapMap), and Native Americans (including Indigenous individuals from the HGDP, Pima, and Mayan individuals) as reference populations. For African Americans, we used a two-population model using ancestral frequencies from Europeans and West Africans (CEU and YRI).

**Supplemental References**

S1. Alexander DH, Lange K. Enhancements to the ADMIXTURE algorithm for individual ancestry estimation. BMC Bioinformatics. 2011;12:246.
