# Supplementary material for: Differential asthma odds following respiratory infection in children from three minority populations
Source: PLoS One. 2020 May 5;15(5):e0231782. doi: 10.1371/journal.pone.0231782 (PMC7199930; doi:10.1371/journal.pone.0231782)
Supplement: S1 Table — Definition of Abbreviations: URI = Upper Respiratory Infection. (DOCX) [file pone.0231782.s003.docx]

**S1 Table.** Pairwise comparison of odds ratios for asthma after two years of age following early-life respiratory infection by racial/ethnic group.

| **Illness** | **Group** | **Group Pair** | | |
| --- | --- | --- | --- | --- |
|  |  | *African American* | *Total Population* | *Mexican* |
| *URI* | *Total Population* | 0.760 | - | - |
|  | *Mexican* | 0.067 | 0.066 | - |
|  | *Puerto Rican* | 0.797 | 0.487 | 0.031 |
| *Pneumonia* | *Total Population* | 0.428 | - | - |
|  | *Mexican* | 0.882 | 0.578 | - |
|  | *Puerto Rican* | 0.037 | 0.089 | 0.059 |
| *Bronchitis* | *Total Population* | 0.081 | - | - |
|  | *Mexican* | 0.356 | 0.382 | - |
|  | *Puerto Rican* | 0.011 | 0.168 | 0.057 |
| *Bronchiolitis/RSV* | *Total Population* | 0.435 | - | - |
|  | *Mexican* | 0.733 | 0.147 | - |
|  | *Puerto Rican* | 0.309 | 0.623 | 0.085 |
| *Any Listed* | *Total Population* | 0.581 | - | - |
|  | *Mexican* | 0.443 | 0.136 | - |
|  | *Puerto Rican* | 0.125 | 0.179 | 0.019 |
