# Supplementary material for: Differential asthma odds following respiratory infection in children from three minority populations
Source: PLoS One. 2020 May 5;15(5):e0231782. doi: 10.1371/journal.pone.0231782 (PMC7199930; doi:10.1371/journal.pone.0231782)
Supplement: S2 Table — (DOCX) [file pone.0231782.s004.docx]

**S2 Table.** Descriptive statistics for selected characteristics of the study population including those diagnosed with asthma before age two (N = 3,722).

|  | **Puerto Rican** | | | | **Mexican American** | | | | **African American** | | | | **Total Population** | | | |
| --- | --- | --- | --- | --- | --- | --- | --- | --- | --- | --- | --- | --- | --- | --- | --- | --- |
|  | ***Case*** | | ***Control*** | | ***Case*** | | ***Control*** | | ***Case*** | | ***Control*** | | ***Case*** | | ***Control*** | |
|  | *N* | *%* | *N* | *%* | *N* | *%* | *N* | *%* | *N* | *%* | *N* | *%* | *N* | *%* | *N* | *%* |
| Number of subjects | 791 | 100 | 783 | 100 | 499 | 100 | 504 | 100 | 699 | 100 | 446 | 100 | 1989 | 100 | 1733 | 100 |
| Males | 439 | 55.5 | 361 | 46.1 | 288 | 57.7 | 210 | 41.7 | 370 | 52.9 | 187 | 41.9 | 1097 | 55.2 | 758 | 43.7 |
| Underweight at birth | 84 | 10.6 | 61 | 7.8 | 39 | 7.8 | 35 | 6.9 | 73 | 10.4 | 45 | 10.1 | 196 | 9.9 | 141 | 8.1 |
| In-utero smoke exposure | 53 | 6.7 | 40 | 5.1 | 17 | 3.4 | 9 | 1.8 | 139 | 19.9 | 54 | 12.1 | 209 | 10.5 | 103 | 5.9 |
| Breastfed | 428 | 54.1 | 432 | 55.2 | 379 | 76 | 401 | 79.6 | 399 | 57.1 | 244 | 54.7 | 1206 | 60.6 | 1077 | 62.1 |
| Number of older siblings |  |  |  |  |  |  |  |  |  |  |  |  |  |  |  |  |
| *0* | 197 | 24.9 | 200 | 25.5 | 169 | 33.9 | 195 | 38.7 | 393 | 56.2 | 208 | 46.6 | 759 | 38.2 | 603 | 34.8 |
| *1* | 240 | 30.3 | 274 | 35 | 185 | 37.1 | 176 | 34.9 | 161 | 23 | 132 | 29.6 | 586 | 29.5 | 582 | 33.6 |
| *2 or more* | 354 | 44.8 | 309 | 39.5 | 145 | 29.1 | 133 | 26.4 | 145 | 20.7 | 106 | 23.8 | 644 | 32.4 | 548 | 31.6 |
| Socioeconomic status* |  |  |  |  |  |  |  |  |  |  |  |  |  |  |  |  |
| *high* | 232 | 29.3 | 271 | 34.6 | 142 | 28.5 | 116 | 23 | 240 | 34.3 | 160 | 35.9 | 614 | 30.9 | 547 | 31.6 |
| *medium* | 142 | 18 | 136 | 17.4 | 104 | 20.8 | 84 | 16.7 | 183 | 26.2 | 137 | 30.7 | 429 | 21.6 | 357 | 20.6 |
| *low* | 417 | 52.7 | 376 | 48 | 253 | 50.7 | 304 | 60.3 | 276 | 39.5 | 149 | 33.4 | 946 | 47.6 | 829 | 47.8 |
| Recruitment site |  |  |  |  |  |  |  |  |  |  |  |  |  |  |  |  |
| *Chicago* | 36 | 4.6 | 23 | 2.9 | 178 | 35.7 | 180 | 35.7 | - | - | - | - | 214 | 10.8 | 203 | 11.7 |
| *Houston* | 2 | 0.3 | - | - | 126 | 25.3 | 91 | 18.1 | - | - | - | - | 128 | 6.4 | 91 | 5.3 |
| *New York* | 64 | 8.1 | 34 | 4.3 | 28 | 5.6 | 64 | 12.7 | - | - | - | - | 92 | 4.6 | 98 | 5.7 |
| *San Francisco Bay Area* | 1 | 0.1 | 1 | 0.1 | 167 | 33.5 | 169 | 33.5 | 699 | 100 | 446 | 100 | 867 | 43.6 | 616 | 35.5 |
| *Puerto Rico* | 688 | 87 | 725 | 92.6 | - | - | - | - | - | - | - | - | 688 | 34.6 | 725 | 41.8 |
| URI | 254 | 32.1 | 35 | 4.5 | 55 | 11 | 27 | 5.4 | 200 | 28.6 | 16 | 3.6 | 509 | 25.6 | 78 | 4.5 |
| Pneumonia | 71 | 9 | 5 | 0.6 | 49 | 9.8 | 11 | 2.2 | 64 | 9.2 | 14 | 3.1 | 184 | 9.3 | 30 | 1.7 |
| Bronchitis | 204 | 25.8 | 10 | 1.3 | 63 | 12.6 | 10 | 2 | 53 | 7.6 | 6 | 1.3 | 320 | 16.1 | 26 | 1.5 |
| Bronchiolitis/RSV | 162 | 20.5 | 14 | 1.8 | 8 | 1.6 | 4 | 0.8 | 29 | 4.1 | 2 | 0.4 | 199 | 10 | 20 | 1.2 |
| Any Listed | 419 | 53 | 54 | 6.9 | 133 | 26.7 | 46 | 9.1 | 264 | 37.8 | 31 | 7 | 816 | 41 | 131 | 7.6 |

*Socioeconomic status was derived from a combination of mother’s education level, health insurance status, and household income weighted by region, see **S1 Text** for more information.
