# Supplementary material for: Differential asthma odds following respiratory infection in children from three minority populations
Source: PLoS One. 2020 May 5;15(5):e0231782. doi: 10.1371/journal.pone.0231782 (PMC7199930; doi:10.1371/journal.pone.0231782)
Supplement: S3 Table — Definition of Abbreviations: URI = Upper Respiratory Infection. (DOCX) [file pone.0231782.s005.docx]

**S3 Table.** Adjusted odds ratios and confidence intervals from the population-specific and combined association analysis between respiratory illnesses in the first two years of life and physician-diagnosed asthma according to race/ethnicity and in the total population in GALA II and SAGE: 2006-2014.

|  | **Puerto Rican (n=1,574)** | **Mexican American (n=1,002)** | **African American (n=1,145)** | **Total Population (n=3,721)** |
| --- | --- | --- | --- | --- |
| URI | 10.60 (7.35-15.60) | 3.71 (2.17-6.59) | 10.20 (6.19-17.90) | 8.45 (6.5-11.10) |
| Pneumonia | 15.30 (6.77-44.0) | 4.25 (2.23-8.82) | 3.07 (1.73-5.82) | 5.38 (3.66-8.20) |
| Bronchitis | 29.40 (16.20-60) | 6.56 (3.43-13.9) | 5.42 (2.47-14.30) | 14.70 (9.8-23.10) |
| Bronchiolitis/RSV | 15.20 (8.98-27.80) | 1.75 (0.53-6.77) | 10.0 (2.96-62.70) | 12.20 (7.56-21.0) |
| Any Listed | 16.10 (11.8-22.20) | 4.39 (2.98-6.60) | 7.79 (5.31-11.80) | 9.68 (7.84-12.0) |

*Adjusted for sex, underweight at birth, maternal smoking during pregnancy, breastfeeding, number of older siblings, SES, recruitment site and global ancestry. Recruitment site was not included in the African American models as they were recruited from one site only.
