# Supplementary material for: Differential asthma odds following respiratory infection in children from three minority populations
Source: PLoS One. 2020 May 5;15(5):e0231782. doi: 10.1371/journal.pone.0231782 (PMC7199930; doi:10.1371/journal.pone.0231782)
Supplement: S4 Table — (DOCX) [file pone.0231782.s006.docx]

**S4 Table.** Pairwise comparison of odds ratios for asthma following early-life respiratory infection by racial/ethnic group including those diagnosed with asthma before age two.

| **Illness** | **Group** | **Group Pair** | | |
| --- | --- | --- | --- | --- |
|  |  | *African American* | *Combined* | *Mexican* |
| *URI* | *Combined* | 0.537 | - | - |
|  | *Mexican* | 0.010 | 0.009 | - |
|  | *Puerto Rican* | 0.913 | 0.344 | 0.002 |
| *Pneumonia* | *Combined* | 0.129 | - | - |
|  | *Mexican* | 0.483 | 0.561 | - |
|  | *Puerto Rican* | 0.004 | 0.040 | 0.028 |
| *Bronchitis* | *Combined* | 0.042 | - | - |
|  | *Mexican* | 0.735 | 0.051 | - |
|  | *Puerto Rican* | 0.002 | 0.080 | 0.002 |
| *Bronchiolitis/RSV* | *Combined* | 0.802 | - | - |
|  | *Mexican* | 0.073 | 0.004 | - |
|  | *Puerto Rican* | 0.601 | 0.572 | 0.002 |
| *Any Listed* | *Combined* | 0.347 | - | - |
|  | *Mexican* | 0.045 | 0.001 | - |
|  | *Puerto Rican* | 0.005 | 0.009 | 0.000 |
