# Supplementary material for: Differential asthma odds following respiratory infection in children from three minority populations
Source: PLoS One. 2020 May 5;15(5):e0231782. doi: 10.1371/journal.pone.0231782 (PMC7199930; doi:10.1371/journal.pone.0231782)
Supplement: S5 Table — (DOCX) [file pone.0231782.s007.docx]

**S5 Table.** Descriptive characteristics of Puerto Rican Islanders and Mainlanders.

|  | **Islander** | | | | **Mainlander** | | | |
| --- | --- | --- | --- | --- | --- | --- | --- | --- |
|  | ***Case*** | | ***Control*** | | ***Case*** | | ***Control*** | |
|  | *N* | *%* | *N* | *%* | *N* | *%* | *N* | *%* |
| Number of subjects | 237 | 100 | 725 | 100 | 47 | 100 | 58 | 100 |
| Males | 127 | 53.6 | 333 | 45.9 | 23 | 48.9 | 28 | 48.3 |
| Underweight at birth | 28 | 11.8 | 57 | 7.9 | 4 | 8.5 | 4 | 6.9 |
| In-utero smoke exposure | 7 | 3 | 33 | 4.6 | 7 | 14.9 | 7 | 12.1 |
| Breastfed | 132 | 55.7 | 410 | 56.6 | 9 | 19.1 | 22 | 37.9 |
| Number of older siblings |  |  |  |  |  |  |  |  |
| *0* | 55 | 23.2 | 179 | 24.7 | 19 | 40.4 | 21 | 36.2 |
| *1* | 75 | 31.6 | 252 | 34.8 | 17 | 36.2 | 22 | 37.9 |
| *2 or more* | 107 | 45.1 | 294 | 40.6 | 11 | 23.4 | 15 | 25.9 |
| Socioeconomic status* |  |  |  |  |  |  |  |  |
| *high* | 80 | 33.8 | 253 | 34.9 | 9 | 19.1 | 18 | 31 |
| *medium* | 46 | 19.4 | 128 | 17.7 | 5 | 10.6 | 8 | 13.8 |
| *low* | 111 | 46.8 | 344 | 47.4 | 33 | 70.2 | 32 | 55.2 |
| Recruitment site |  |  |  |  |  |  |  |  |
| *Chicago* | - | - | - | - | 18 | 38.3 | 23 | 39.7 |
| *Houston* | - | - | - | - | 1 | 2.1 | - | - |
| *New York* | - | - | - | - | 27 | 57.4 | 34 | 58.6 |
| *San Francisco Bay Area* | - | - | - | - | 1 | 2.1 | 1 | 1.7 |
| *Puerto Rico* | 237 | 100 | 725 | 100 | - | - | - | - |
| URI | 50 | 21.1 | 24 | 3.3 | 7 | 14.9 | 11 | 19 |
| Pneumonia | 12 | 5.1 | 4 | 0.6 | 1 | 2.1 | 1 | 1.7 |
| Bronchitis | 35 | 14.8 | 9 | 1.2 | 1 | 2.1 | 1 | 1.7 |
| Bronchiolitis/RSV | 28 | 11.8 | 13 | 1.8 | 3 | 6.4 | 1 | 1.7 |

*Socioeconomic status was derived from a combination of mother’s education level, health insurance status, and household income weighted by region, see **Text A in S1 File** for more information.
