# Supplementary material for: Differential asthma odds following respiratory infection in children from three minority populations
Source: PLoS One. 2020 May 5;15(5):e0231782. doi: 10.1371/journal.pone.0231782 (PMC7199930; doi:10.1371/journal.pone.0231782)
Supplement: S6 Table — Definition of Abbreviations: URI = Upper Respiratory Infection. (DOCX) [file pone.0231782.s008.docx]

**S6 Table.** Odds ratios and confidence intervals from the geography-specific analysis between respiratory illnesses in the first two years of life and physician-diagnosed asthma after the age of two in the two Puerto Rican populations: Islanders and Mainlanders.

|  | **Islander (n=962)** | **Mainlander (n=102)** |
| --- | --- | --- |
| URI | 7.80 (4.69-13.30) | 0.82 (0.25-2.57) |
| Pneumonia | 8.92 (3.04-32.40) | 1.06 (0.04-29.80) |
| Bronchitis | 14.80 (7.23-33.40) | 0.63 (0.02-18.60) |
| Bronchiolitis/RSV | 7.60 (3.9-15.60) | 3.86 (0.39-91.30) |
| Any Listed | 8.64 (5.72-13.20) | 0.68 (0.22-2.01) |

*Adjusted for sex, underweight at birth, maternal smoking during pregnancy, breastfeeding, number of older siblings, SES, recruitment site and global ancestry. Recruitment site was not included in the African American models as they were recruited from one site only.
