# Supplementary material for: Differential asthma odds following respiratory infection in children from three minority populations
Source: PLoS One. 2020 May 5;15(5):e0231782. doi: 10.1371/journal.pone.0231782 (PMC7199930; doi:10.1371/journal.pone.0231782)
Supplement: Attachment — Submitted filename: Plos_review_RSV_PR.docx [file pone.0231782.s009.docx]

The authors present an analysis looking at the relationship between ethnicity and asthma, using 2006 through 2014 data from two case-control studies (GALA II and SAGE). The authors report that the odds of asthma were higher among Puerto Ricans (from the island of Puerto Rico, not the mainland USA) than the odds observed among Mexican Americans and African Americans.

This study is overall interesting and has scientific merit. There are several issues that the authors need to address and clarifications that are needed, in order to better understand the findings. One big limitation of the study is that it relies upon self-reporting of history of respiratory infections as well as diagnosis of asthma, both of which are subject to recall bias among other biases.

**Abstract**

- There is no explanation of which are the other ethnic groups studied and this should be included.
- Line 77 should state “odds were” not “odds was”

**Introduction**

- It is unusual to include a figure with results in the introduction; please move this figure to the results if these are indeed results.
- In Line 119, please clarify that the Puerto Ricans studied are not mainland but rather are from the island.

**Methods**

- Lines 136 through 142 detail a combination of methods and results; please separate and provide the results (i.e., the number excluded) in the results section separate from the methodological criteria (i.e., exclusion criteria) in the methods
- It is unclear if the diagnosis of asthma was confirmed somehow or if this is simply self-reporting by the patient that a doctor told them that they had asthma; please clarify what “physician-diagnosed asthma” means (Line 144)
- Was there any matching of the cases and the controls?
- More details are needed about the logistic regression model and process used to assess confounders and effect measure modifiers
- What was the case definition for each clinical outcome?

**Results**

- How were the clinical outcomes defined for participants (i.e., most people will not know what bronchiolitis is or a URI)
- Risk is used throughout the manuscript when the authors are assessing odds. Odds and risk can only be used interchangeably when the outcome is rare; please address this throughout the manuscript.
- Prevalence and incidence are used in various places to refer to incidence; please address this throughout the manuscript

**Discussion**

- Have you compared your self-reported asthma prevalence to what is reported through the BRFSS? Please include those data if they are available in the Discussion.
- Lines 241-242 and 270-271 are bolded and in a different font; is this an error?
- Throughout the Discussion, there is discussion of relative risk; I didn’t an analysis of risk presented; please address correct this to state odds
- Line 301 has a typo with a recurrent use of the word “Islanders”; please correct
- There is a big issue with recall bias that affects the potential validity of the results; this needs to be discussed in more detail
- This issue with recall bias is especially problematic if there is an ethnic specific pattern to this; what does the literature show about this?
- Please include a discussion of other studies that have shown limited relationship between RSV and subsequent asthma

<https://www.ncbi.nlm.nih.gov/pubmed/3137006>

**Figure 1**

- What is the source of these RSV data? There is no description in the methods of where these data come from; please address this

**Figure 2**

- Only <10% of parents reported that their children had any URI in the first two years of life; this seems very low. Please provide estimates from the literature to support that these numbers are comparable to what has been found in other studies that do not rely on self-reporting.
