# Supplementary material for: Differential asthma odds following respiratory infection in children from three minority populations
Source: PLoS One. 2020 May 5;15(5):e0231782. doi: 10.1371/journal.pone.0231782 (PMC7199930; doi:10.1371/journal.pone.0231782)
Supplement: Attachment — Submitted filename: Response_to_Reviewers PLOS One.docx [file pone.0231782.s010.docx]

March 05, 2020

Sreekumar Othumpangat, PhD
Academic Editor
PLOS ONE

**PONE-D-19-32435: Differential asthma risk following respiratory infection in children from three minority populations**

Dear Dr. Othumpangat,

We would like to thank the Editor and the Reviewers for their thoughtful comments and appreciate the opportunity to resubmit a revised manuscript addressing the comments. Line numbers correspond to the “Marked Up” version of the manuscript (MS). If you believe that any detail was omitted during the revision or any further information is needed, please do not hesitate to let us know.

**Response to reviewers**

**Editor comments**

In this manuscript authors address whether early life respiratory illness was associated with later life asthma in minority children. There are several concerns that need to be addressed as stated by reviewer 1 and 2.

C1. **(A)** Authors need to add more information on bronchiolitis of RSV and HRV. **(B)** It is not clear whether the self-reported asthma or diagnosed by a physician before the age of 2 from the methods presented.

**We have labeled the Editor’s comments (A and B) and address them with labeled responses below:**

**(A) Additional information on RSV vs HRV bronchiolitis has been added to the introduction and methods. Specifically, in the introduction, we included the early-age predominance of RSV which then changes to HRV over time, the quantification of the relative risk of asthma after RSV and HRV, and the statement that bronchiolitis is a clinical diagnosis (Lines 126 – 128). To the methods we added that we did not use pathogen identification in this retrospective study for RSV.**

**“*As this was a retrospective study, we could not rely on pathogen identification as a means to confirm the viral species present during the illness.”* (Lines 190 – 192).**

**(B) We apologize for any confusion and have updated the text to clarify that asthma was physician diagnosed reported by the parent.**

**“*The outcome of our study was a physician diagnosed asthma after the age of two reported by the parent*” (Lines 181 – 182)**

C2. In results the Puerto Ricans were more likely to report RSV infection than the other minorities, does this has anything to do with the genetics or is it fully related to the earlier infection?

**We thank the Editor for their question. However, we feel that the answer lies beyond the scope of our project. A prospective birth cohort study is needed to assess if report of RSV is higher in Puerto Ricans because they are genetically predisposed to RSV; if earlier infections alter the airway which can lead to worse airway infections like RSV, or if it is simply because they live in an area where RSV infection has a year-round seasonality. Our lab is currently funded for a birth cohort in Puerto Rico to answer such questions, with recruitment starting in March 2020.**

C3. Not much information provided with the early life HRV, does the data show a similar effect as RSV?

**We have added information in the manuscript relating to HRV bronchiolitis, including the early-life predominance of RSV, which transitions to HRV at some point after 1 year of age. We also added the relative risk of asthma after RSV and HRV to the introduction to clarify these relationships (Lines 126 – 128).**

C4. Discussion may address other factors like environment, genetic variation or life style that could have a role in mainlanders and the islanders.

**We thank the Editor for their comment and have added to the discussion that other factors, such as environment and socio-cultural factors, could also affect asthma risk in Islanders versus Mainlanders.**

***“Respiratory infections likely represent one of several factors leading to increased asthma incidence in Islander Puerto Ricans relative to Mainlanders, which may include other environmental and socio-cultural factors.”* (Lines 422 – 425)**

C5. Line 298 to 302, modify the sentences, to avoid confusion on mainlanders and islanders.

**We appreciate the Editor for their keen eye and have corrected the text in the manuscript to avoid confusion:**

**“…*we find it interesting that Mainlander Puerto Ricans tended to have lower odds of asthma after respiratory infections relative to Islanders.*” (Lines 419 – 421)**

C6. Please ensure that your manuscript meets PLOS ONE's style requirements, including those for file naming. The PLOS ONE style templates can be found at
    <http://www.journals.plos.org/plosone/s/file?id=wjVg/PLOSOne_formatting_sample_main_body.pdf> and <http://www.journals.plos.org/plosone/s/file?id=ba62/PLOSOne_formatting_sample_title_authors_affiliations.pdf>

**We apologize for any oversight and have made sure all parts of the manuscript meet PLOS One’s style requirements.**

C7. Please provide additional details regarding participant consent. In the ethics statement in the Methods and online submission information, please ensure that you have specified whether consent was written or verbal/oral. If consent was verbal/oral, please specify: 1) whether the ethics committee approved the verbal/oral consent procedure, 2) why written consent could not be obtained, and 3) how verbal/oral consent was recorded. If your study included minors, please state whether you obtained consent from parents or guardians in these cases.

**We thank the editor for their concern and have clarified that consent was written and was either obtained from all adult participants (age ≥18) or the parent/legal guardians of minor participants.**

**“*All participants provided written consent to being in the study. Consent was obtained from all adult participants and parent/legal guardians of minor participants*” (Lines 164 – 166)**

C8. Thank you for including your ethics statement: *"All participating institutions obtained the appropriate approvals from their Institutional Review Boards."* Please amend your current ethics statement to include the full name of the ethics committee/institutional review board(s) that approved your specific study.

**We have modified our current ethics statement to include the requested information:**

**“*The study protocols for both GALA II and SAGE were approved by the UCSF Human Research Protection Program Institutional Review Board (IRB) and all institutions participating in recruitment obtained the appropriate approvals from their IRBs for recruitment related activity.*” (Lines 166 – 169)**

C9. We note that you have indicated that data from this study are available upon request. PLOS only allows data to be available upon request if there are legal or ethical restrictions on sharing data publicly. For information on unacceptable data access restrictions, please see http://journals.plos.org/plosone/s/data-availability#loc-unacceptable-data-access-restrictions. In your revised cover letter, please address the following prompts:
    a. If there are ethical or legal restrictions on sharing a de-identified data set, please explain them in detail (e.g., data contain potentially identifying or sensitive patient information) and who has imposed them (e.g., an ethics committee). Please also provide contact information for a data access committee, ethics committee, or other institutional body to which data requests may be sent.
    b. If there are no restrictions, please upload the minimal anonymized data set necessary to replicate your study findings as either Supporting Information files or to a stable, public repository and provide us with the relevant URLs, DOIs, or accession numbers. Please see http://www.bmj.com/content/340/bmj.c181.long for guidelines on how to de-identify and prepare clinical data for publication. For a list of acceptable repositories, please see <http://journals.plos.org/plosone/s/data-availability#loc-recommended-repositories>.

**We thank the Editor for clarifying the data availability requirement. However, as stated, we feel that due to the sensitive nature of the data collected surrounding issues of race/ethnicity, socioeconomic status, and ancestry, our study qualifies for this requirement to be waived. Additionally, our group has previously published in PLOS One using the same reasoning as presented in this manuscript.** [**https://journals.plos.org/plosone/article?id=10.1371/journal.pone.0179091**](https://journals.plos.org/plosone/article?id=10.1371/journal.pone.0179091)

C10. Please include captions for your Supporting Information files at the end of your manuscript, and update any in-text citations to match accordingly. Please see our Supporting Information guidelines for more information: <https://journals.plos.org/plosone/s/supporting-information>.

**We appreciate the Editor for supplying us with a guide to properly format the Supporting Information. We have included captions as the Editor has requested in line with the style guide.**

**Reviewer 1 comments**

**Abstract**

C11. There is no explanation of which are the other ethnic groups studied and this should be included.

**We thank the Reviewer for her/his observation and have revised the methods section in our abstract to specify that we performed this study in Puerto Rican, Mexican American, and African American children. We have also clarified this issue throughout the MS.**

C12. Line 77 should state “odds were” not “odds was”.

**The term as is is correct. Odds is noun, plural in form but singular or plural in construction (https://www.merriam-webster.com/dictionary/odds). Thus, we will prefer to keep as singular for consistency with research methods language. No change was made to the MS.**

**Introduction**

C13. It is unusual to include a figure with results in the introduction; please move this figure to the results if these are indeed results.

**We thank the Reviewer for her/his comment. However, Figure 1 is not results but rather a simplified representation of the results obtained from** [**McGuinness et al. Pediatr Infect Dis J. 2014**](https://www.ncbi.nlm.nih.gov/pubmed/24445835) **to illustrate that RSV season is a year round in Puerto Rico. We believe it is an important figure to include in our main manuscript because it highlights the drastic differences in RSV rates between the Mainland United States and the Island of Puerto Rico. We have updated the figure caption to clarify the source of this data:**

**“*Data shown are a simplified representation of the data obtained from McGuinness et al. Pediatr Infect Dis J. 2014.[29]*” (Lines 137 – 138)**

C14. In Line 119, please clarify that the Puerto Ricans studied are not mainland but rather are from the island.

**We thank the Reviewer for her/his input and apologize for any confusion. Both Mainland and Islander Puerto Ricans were included in the main analysis. No change was made to the MS.**

**Methods**

C15. Lines 136 through 142 detail a combination of methods and results; please separate and provide the results (i.e., the number excluded) in the results section separate from the methodological criteria (i.e., exclusion criteria) in the methods.

**We appreciate the Reviewer’s concern. However, the text does not present results. In fact, the text describes the original sample and the exclusions to arrive at the analytical sample. No change was made to the MS.**

C16. It is unclear if the diagnosis of asthma was confirmed somehow or if this is simply self-reporting by the patient that a doctor told them that they had asthma; please clarify what “physician-diagnosed asthma” means (Line 144).

**We respectfully direct the Reviewer to response Comment 1B.**

C17. Was there any matching of the cases and the controls?

**We thank the Reviewer’s comment. However, there was no case-control matching in our analysis. No change was made to the MS.**

C18. More details are needed about the logistic regression model and process used to assess confounders and effect measure modifiers.

**We have updated the methods surrounding the logistic regression model and assessment of confounders and effect modifiers as per the Reviewer requested. Specifically, we updated our *Covariate* and *Statistical Analysis* sections in Methods to the following:**

**“*Covariates***

***Consistent with previous studies,[32, 33] we considered factors known to be associated with early-life respiratory illnesses and with asthma. These included sex, underweight at birth (yes/no), maternal smoking during pregnancy (yes/no), whether the subject was breastfed (yes/no), number of older siblings (none, one, two or more), socioeconomic status (SES; low, medium, high; described further in S1 Text), and recruitment region. However, recruitment site was not included in the African American models because all the participants were recruited from the same region.***

***Statistical Analysis***

***Descriptive statistics were calculated for the overall study population and for each minority population separately. We used logistic regression to quantify the association between physician-diagnosed asthma and self-reported respiratory illnesses in the first two years of life in all three of our populations (Puerto Ricans, Mexican Americans, and African Americans) combined. We adjusted for the underlying substructure of populations in the dataset using global African and European ancestry estimates (described further in S2 Text). Because race/ethnicity may act as an effect modifier due to the large differences in asthma prevalence,[6] we estimated this association stratified by race/ethnicity. Significance for main effects was determined at* p *< 0.05. The statistical programming language R version 3.5.1 was used to perform all analyses.*” (Lines 193 – 218)**

C19. What was the case definition for each clinical outcome?

**Clinical outcomes were defined by a physician diagnosis of the clinical outcome reported by the parent. The following is an exact replication of the questions asked in our questionnaire that we used to determine a yes/no report of a clinical outcome:**

**Was [CHILD] seen by a doctor or other health care provider for a chest illness *before* the age of 2 years?**

**[ ] Yes	[ ] No**

**What was the diagnosis? (*read all and check all that apply*)**

i. **Pneumonia	[ ] Yes**
ii. **Bronchitis	[ ] Yes**
iii. **Reactive airway disease	[ ] Yes**
iv. **Bronchiolitis or RSV	[ ] Yes**
v. **Croup	[ ] Yes**
vi. **Asthma	[ ] Yes**
vii. **Cold	[ ] Yes**
viii. **Other	[ ] Yes**
ix. **Don’t Know	[ ] Yes**

**Results**

C20. How were the clinical outcomes defined for participants (i.e., most people will not know what bronchiolitis is or a URI).

**Please see our response to the previous Comment #19 as to how clinical outcomes were defined. To elaborate further, any questions by participants regarding the definition of clinical outcomes was clarified by recruitment staff with clinical expertise at the time of questionnaire administration.**

C21. Risk is used throughout the manuscript when the authors are assessing odds. Odds and risk can only be used interchangeably when the outcome is rare; please address this throughout the manuscript.

**We thank the Reviewer for pointing this out. We have changed risk to odds where appropriate throughout the MS.**

C22. Prevalence and incidence are used in various places to refer to incidence; please address this throughout the manuscript.

**Again, we thank the Reviewer for her/his insight and have changed prevalence to incidence where appropriate throughout the MS.**

**Discussion**

C23. Have you compared your self-reported asthma prevalence to what is reported through the BRFSS? Please include those data if they are available in the Discussion.

**We thank the Reviewer for this suggestion. However, BRFSS reports total adult prevalence. Since our study is limited to children, we cannot make a direct comparison with the BRFSS data.**

C24. Lines 241-242 and 270-271 are bolded and in a different font; is this an error?

**We appreciate the Reviewer for raising this concern and apologize for any confusion. These are subheadings in our discussion. We have removed them from the MS to avoid further confusion.**

C25. Throughout the Discussion, there is discussion of relative risk; I didn’t an analysis of risk presented; please address correct this to state odds

**We thank their Reviewer for her/his input and have made the appropriates changes.**

C26. Line 301 has a typo with a recurrent use of the word “Islanders”; please correct.

**We have revised the MS as per Comment 7 above.**

C27. There is a big issue with recall bias that affects the potential validity of the results; this needs to be discussed in more detail.

**We thank the Reviewer for this concern. We have addressed recall bias in detail in the *Strengths and limitations* section of our Discussion.**

**“*Specifically, recall bias may affect the accuracy of answers to questions about events in the distant past. However, if bias were to occur, this bias will be non-differential and affect all racial/ethnic groups studied, biasing the results towards the null.*” (Lines 449 – 452)**

C28. This issue with recall bias is especially problematic if there is an ethnic specific pattern to this; what does the literature show about this?

**Please see our response to the previous Comment 27.**

C29. Please include a discussion of other studies that have shown limited relationship between RSV and subsequent asthma. <https://www.ncbi.nlm.nih.gov/pubmed/3137006>

**We have added more information on the relationship between RSV and subsequent asthma in the introduction (line 126 – 128). Please note that the reference provided above was not included as it does not relate to or address RSV and/or asthma.**

**Figure 1**

C30. What is the source of these RSV data? There is no description in the methods of where these data come from; please address this.

**We have addressed this issue as part of Comment 13.**

**Figure 2**

C31. Only <10% of parents reported that their children had any URI in the first two years of life; this seems very low. Please provide estimates from the literature to support that these numbers are comparable to what has been found in other studies that do not rely on self-reporting.

**Although URI is a common reason for presentation to medical care, the vast majority of patients with URIs do not present to medical care. In one retrospective study of adults, only 1% of patients with URI presented to care ([Vingilis et al., Can Fam Physician 1999](https://www.ncbi.nlm.nih.gov/pmc/articles/PMC2328659/)). The rate of URI in children is known to be higher than in adults, and some parents may be more likely to bring a child to care than an adult. However, we do not expect these rates to differ significantly. Therefore, we may surmise that the low rate of physician diagnosis of URI in our patient population may be accurate. A prospective study measuring the rate of physician visits for URI in children and adults found an overall rate of about 0.25 visits per person per year, which over a two-year period could be interpreted as about a 50% risk of URI visit – a number which was further reduced by patient education surrounding URI (**[**Roberts et al., JAMA 1983**](https://jamanetwork.com/journals/jama/article-abstract/388311)**). With some element of recall bias related to mild illness, as discussed above, we feel that our data are consistent with what has previously been described.**

**Reviewer 2 comments**

C32. In this work, the authors explored the association between early-life respiratory illness and physician-diagnosed asthma at 2 years in three minority populations and found a strong association. Though the findings are not new, it has strengthened existing evidence. The authors need to clearly mention the value of the findings and specific recommendations for the next step.

**We thank the Reviewer for pointing this out and have added the racial/ethnic variability of asthma risk after respiratory infection and the especially high odds for Puerto Rican children as a novel finding to our discussion (Lines 392 – 395).**

C33. It would be worth to mention in the background some hypothesis citing previous relevant work why asthma morbidity and mortality has been strikingly higher among Puerto Rican as early-life respiratory infections are one of many drivers that contributes asthma development.

**We thank the Reviewer for their comment and have updated the text in our introduction to further explain why Puerto Ricans may be uniquely burdened beyond the risks imposed by early-life respiratory illness (Lines 117 – 121). In addition, we have added a hypothesis statement at the end of the introduction (Lines 147 – 150).**

C34. It is not clear whether the etiologies of respiratory illnesses (URI, pneumonia, bronchitis) were investigated. Most published works so far reportedly linked early life viral respiratory infections with childhood asthma. If the etiologic data is available it would be good to perform a sub-analysis to look for specific association between viral URI/pneumonia before two years and asthma at two years of age and how this varies by ethnicity.

**We appreciate the Reviewer’s comment and see the value of examining such association. However, the sub-analysis is beyond the scope of this MS and not possible based on the retrospective nature of the study.**

C35. Line 151: It is not clear whether all bronchiolitis episodes had a detectable RSV, although we know that most bronchiolitis is caused by RSV.

**We appreciate the Reviewer’s comment and agree that the majority of bronchiolitis is caused by RSV. A minority of patients diagnosed with bronchiolitis may also be caused by HRV or others viral infections however, the retrospective nature of the study did not allow for organism identification during respiratory illnesses. We have updates in the “Exposures” section of our MS to clarify this point. (Lines 190 – 192)**

C36. Line 154: atopic status (this might vary by ethnicity) of enrolled children needed to be addressed as a covariate as this might influence the relationship between respiratory infections and risk of asthma development <https://www.sciencedirect.com/science/article/abs/pii/S0091674907002382>.

**We acknowledge the Reviewer’s concern. However, after performing additional analyses, we believe that atopic status should not be considered as a covariate in our main analysis. Specifically, because atopic dermatitis and atopic rhinitis were exclusion criteria from our study populations (GALA II and SAGE) for controls, we defined atopy by total immunoglobulin E (IgE) levels. In our populations, serum IgE is elevated in Puerto Ricans in general, so including this covariate in our combined analysis would severely skew the results. As such, we performed a stratified analysis of atopic (IgE ≥ 100 kU/L) versus non-atopic (IgE < 100 kU/L) participants in our Puerto Rican population only because they constituted our largest population. When comparing participants according to atopic status atopic (IgE ≥ 100 kU/L versus IgE < 100 kU/L), the results (below) from our stratified analysis reported non-significant differences between the models for each illness and only marginal significance if the participant reported having at least one illness. Therefore, we are confident that atopic status would not have affected our main analysis and would prefer not include it as covariate.**

| **Illness** | **p-value*** |
| --- | --- |
| Upper respiratory infection | 0.2287 |
| Pneumonia | 0.9871 |
| Bronchitis | 0.0527 |
| Bronchiolitis/RSV | 0.7624 |
| Any Listed | 0.0499 |

*** p-values were calculated using a t-test**
